# Association of blood urea nitrogen to serum albumin ratio with arterial stiffness in type 2 diabetes patients: a Chinese cross-sectional study

**DOI:** 10.3389/fendo.2025.1663831

**Published:** 2025-10-08

**Authors:** Shengqing He, Wanrui Meng, Miaoling Huang, Qingsong Fu, Jie Shen

**Affiliations:** ^1^ Department of Endocrinology and Metabolism, The Eighth Affiliated Hospital, Southern Medical University (The First People’s Hospital of Shunde), Foshan, Guangdong, China; ^2^ GuangDong Engineering Technology Research Center of Metabolic Disorders Interdisciplinary Precision Prevention and Digital Healthcare, The Eighth Affiliated Hospital of Southern Medical University (The First People’s Hospital of Shunde), Foshan, Guangdong, China; ^3^ Department of Endocrinology, Longgang Central Hospital, Shenzhen, Guangdong, China; ^4^ Department of Laboratory, Longgang Central Hospital, Shenzhen, Guangdong, China

**Keywords:** type 2 diabetes mellitus, arterial stiffness, blood urea nitrogen to serum albumin ratio, brachial-ankle pulse wave velocity, cardiovascular disease

## Abstract

**Background:**

Arterial stiffness is an early indicator of atherosclerosis. The blood urea nitrogen to serum albumin ratio(BAR) is associated with poor prognosis in several chronic diseases. However, the relationship between BAR and arterial stiffness in type 2 diabetes mellitus (T2DM) patients has not been extensively studied. This study aimed to examine the relationship between BAR and brachial-ankle pulse wave velocity (baPWV), an indicator of arterial stiffness in patients newly diagnosed with T2DM.

**Methods:**

A total of 510 adult patients newly diagnosed with T2DM were enrolled between January 2021 and December 2023. BAR was calculated by blood urea nitrogen/albumin ratio. A baPWV ≥1400 cm/s was defined as arterial stiffness. A linear regression model and logistic regression model were used to assess the relationship between BAR and baPWV after adjusting for potential confounders.

**Results:**

The average age of the patients in this study was 45.66 ± 10.18 years, and 78.8% were male. The mean baPWV was 1469.15 ± 295.82 cm/s, and 50.8% of patients exhibited arterial stiffness. The prevalence of arterial stiffness increased significantly across ascending BAR tertiles (T1: 39.3%, T2: 51.7%, T3: 61.4%; *p* = 0.002). Linear correlation analysis revealed a positive correlation between BAR and baPWV. According to the fully adjusted logistic regression model, each unit increase in the lnBAR was associated with a 3.452-fold greater risk of arterial stiffness[95% CI(1.586, 7.513), *p* = 0.002]. Compared to the lowest tertile (T1), participants in the middle (T2) and highest (T3) BAR tertiles had a significantly greater risk of arterial stiffness [T2: OR = 1.915, 95% CI (1.016, 3.609), *p* = 0.044; T3: OR = 2.064, 95% CI (1.051, 4.054), *p* = 0.035]. Stratified analyses demonstrated consistent positive correlations between BAR and baPWV across sex and BMI subgroups, as well as in individuals aged < 50 years.

**Conclusion:**

BAR levels were independently and positively correlated with baPWV in Chinese patients with newly diagnosed T2DM.

## 1 Introduction

The global diabetes epidemic poses a significant challenge to public health systems worldwide, imposing considerable economic and social burdens. According to the International Diabetes Federation (IDF) (1), 589 million adults aged 20–79 years are living with diabetes globally. This number is expected to rise to 853 million by 2050, with over 90% of cases attributed to type 2 diabetes mellitus (T2DM). China has the highest diabetes burden, affecting 12.8% of the adult population (2). Crucially, diabetes-related complications, particularly macrovascular diseases such as atherosclerosis, ischemic heart disease, and stroke, have emerged as the leading causes of mortality and disability among patients with diabetes. Importantly, these complications are increasingly prevalent among younger populations (3), underscoring the urgent need for early screening of high-risk individuals and implementation of targeted vascular protection strategies.

Arterial stiffness, characterized by diminished arterial elasticity due to structural and functional abnormalities of the arterial wall, represents an early indicator of atherosclerosis (4). Substantial evidence indicates that increased arterial stiffness is independently correlated with cardiovascular disease (CVD) and renal impairment, serving as a predictor of cardiovascular morbidity and mortality (4–6). Diabetes exacerbates arterial stiffening through various pathophysiological mechanisms, including insulin resistance, endothelial dysfunction, non-enzymatic glycation, platelet dysfunction, chronic inflammation, and increased oxidative stress (7). In patients with T2DM, early detection of arterial stiffness, in conjunction with biomarker identification, is crucial for mitigating the progression of atherosclerosis. Brachial-ankle pulse wave velocity (baPWV), a noninvasive and cost-effective clinical tool, has a strong ability to predict for cardiovascular risk and all-cause mortality in diabetic patients (8, 9). Notably, assessing baPWV in newly diagnosed with T2DM is of significant clinical importance. Many of these patients have already experienced prolonged hyperglycemia, which initiates the progression of vascular stiffness. Early detection of arterial stiffness in this population provides a critical opportunity for intervention, potentially altering the progression of CVD.

Blood urea nitrogen (BUN), a metabolic byproduct of protein catabolism, serves as a biomarker of renal function, nutritional status, and volume status. It has also been independently associated with CVD mortality risk, establishing its utility in the assessment of CVD prognosis (10, 11). Serum albumin(ALB), a key nutritional indicator, critically mediates substance transport, ligand binding, and maintenance of plasma colloid osmotic pressure. Additionally, it has antioxidant, anti-inflammatory, and endothelial stabilizing functions (12, 13). The blood urea nitrogen to serum albumin ratio (BAR) is an innovative biomarker that combines indicators of BUN and ALB, reflecting both nutritional and inflammatory factors. Studies have demonstrated that it has significant predictive value in assessing the prognosis of various diseases, including pneumonia, chronic heart failure, sepsis, and renal insufficiency (14–17). However, its association with arterial stiffness in patients with T2DM remains unknown. Therefore, this study aimed to investigate the correlation between BAR and arterial stiffness, as determined by baPWV, in individuals newly diagnosed with T2DM.

## 2 Methods

### 2.1 Data source and study population

This was a cross-sectional study. A total of 510 adults with newly diagnosed (< 3 months) with T2DM who were drug-naïve—defined as individuals who had not previously received any treatment with oral hypoglycemic agents or insulin—were enrolled. These patients were under follow-up care at the National Metabolic Management Center (MMC) of Longgang Central Hospital in Shenzhen between January 2021 and December 2023. The MMC is a platform for the standardized diagnosis, management, and follow-up of patients with metabolic diseases. All individuals who met the 1999 WHO diagnostic criteria for T2DM were included in the study. The main exclusion criteria were autoimmune diabetes, secondary diabetes, pregnancy, malignancy, chronic kidney disease (eGFR <60 ml/min), chronic liver disease, coronary artery disease, heart failure, and stroke. The study was conducted in accordance with the principles outlined in the Declaration of Helsinki, and the research protocol received approval from the Ethics Committee of Longgang Central Hospital. Informed consent was obtained from all participants.

### 2.2 Clinical and laboratory data

Face-to-face interviews were conducted with all patients to obtain relevant information, including age, sex, occupation, medical history, and personal habits such as smoking and drinking. The duration of T2DM was calculated from the time of the first diagnosis. Blood pressure was measured via an automated blood pressure cuff with the participant in a seated position on the upper arm. The average value was derived from three consecutive measurements. The subjects’ height and weight were measured in a fasted state on the morning of the study, and the body mass index(BMI) of each individual was calculated via the following formula: weight (kg)/height (m)². Waist circumference was measured at the midpoint between the lower edge of the lowest rib and the upper edge of the hipbone using a non-elastic flexible ruler. The average of the two measurements was calculated. Plasma was collected from the patients in a fasted state eight hours after their overnight stay, and the following indicators were measured via a fully automated biochemical analyzer: fasting blood glucose, fasting C-peptide, glycosylated hemoglobin(HbA_1_c), alanine aminotransferase(ALT), aspartate aminotransferase(AST), gamma-glutamyltransferase(GGT), serum albumin(ALB), serum uric acid, creatinine, blood urea nitrogen(BUN), triglyceride(TG), total cholesterol(TC), HDL cholesterol(HDL-C) and LDL cholesterol(LDL-C). The eGFR was calculated from serum creatinine using by abbreviated MDRD study equation [eGFR (mL/min/1.73 m²) = 186 × (Scr)^-1.154 × (Age)^-0.203 × (0.742 if female)] and included as a continuous variable. BAR(mmol/g) was calculated by the ratio of BUN (mmol/L) to albumin(g/L). The patients were divided into three equal groups according to their BAR level:T1, BAR <0.0874; T2, BAR between 0.0874 and 0.1161; and T3, BAR >0.1161.

### 2.3 Brachial-ankle pulse wave velocity measurement

BaPWV was measured via an automated atherosclerosis testing device (VP-1000; Omron Corporation, Kyoto, Japan). The participants were placed in the supine position and allowed to rest for at least five minutes. Blood pressure cuffs were then attached to the upper arms (brachial arteries) and ankles (posterior tibial arteries) bilaterally. Cardiac leads were also connected to synchronize the pulse waveforms in the cuff arteries. The conduction distance between the arm and ankle was measured, and the conduction time was defined as the interval between the initial rise in the waveforms of the brachial and posterior tibial arteries. The baPWV was calculated by the following formula: baPWV (cm/s) = conduction distance (cm)/conduction time (s). The average baPWV was calculated by averaging the baPWV readings from both sides, and baPWV ≥1400 cm/s was defined as arterial stiffness, as previously reported (18, 19). All operations were performed by technicians who had received uniform technical training, and measurements with poor waveforms were excluded.

### 2.4 Statistical analyses

Data that followed a normal distribution are presented as the means ± standard deviations, while data that did not follow a normal distribution are presented as medians with quartile ranges. Count data are presented as frequencies and percentages (n, %). BaPWV was analyzed as a continuous variable using its raw values (cm/s). In the binary logistic regression analysis, BAR was treated as a continuous variable and was transformed and analyzed in the form of natural logarithm because the raw data was skewed. Patients were grouped into tertiles on the basis of BAR. To evaluate trends, linear regression was applied to continuous variables, whereas chi-square trend tests were used for categorical variables. The assumption of linearity in the logit for these continuous variables in the logistic regression model was confirmed using Box-Tidwell test, while for continuous predictors in linear regression, it was confirmed using the Residuals *vs*. Fitted plot. Pearson or Spearman correlation coefficients were used to examine the relationships between baPWV and other indicators. The assessment of multicollinearity among the relative variables was conducted by calculating the Variance Inflation Factor (VIF), and none were found. A multivariable binary logistic regression model was used to compute odds ratios (ORs) and 95% confidence intervals (CIs) for assessing the independent effects of BAR on baPWV. The Receiver Operating Characteristic (ROC) curve analysis was employed to ascertain the optimal cut-off for the BAR in predicting increased arterial stiffness in individuals newly diagnosed with T2DM. Statistical analyses were performed using SPSS 27.0, and a bilateral *p*-value <0.05 was considered statistically significant.

## 3 Results

### 3.1 Baseline characteristics

The demographic and clinical characteristics of the enrolled patients are shown in **Table 1**. A total of 510 patients with newly diagnosed T2DM were included in this study. The average age of the patients was 45.66 ± 10.18 years. Among these patients, 402 (78.8%) were male and 98 (21.2%) were female. The mean baPWV was 1469.15 ± 295.82 cm/s, and 50.8% of patients exhibited arterial stiffness. The enrolled patients were categorized into three groups according to BAR level: T1, BAR <0.0874; T2, BAR between 0.0874 and 0.1161; and T3, BAR >0.1161. A higher BAR was associated with older age, higher blood creatinine and BUN, and lower BMI, eGFR and ALB. However, no significant differences were observed in sex, SBP, DBP, waist circumference, fasting blood glucose, HbA1c, fasting C-peptide, ALT, AST, GGT, serum uric acid, UACR, TG, TC, LDL-C or HDL-C levels. There were no statistically significant differences in smoking or alcohol consumption among the three groups. The higher BAR group had a greater baPWV. The percentage of patients with arterial stiffness in the T1, T2, and T3 subgroups were 40.6%, 52.4%, and 59.4%, respectively (*p* = 0.002), indicating an increase in the prevalence of arterial stiffness according to BAR subgroup.

**Table 1:** Baseline characteristics of participants according to different BAR level groups.

Variables	ALL	T1 (n=170)	T2 (n=170)	T3 (n=170)	*p-value*
Sex (men) (case, %)	402, 78.8%	131, 77.1%	132, 77.6%	139, 81.1%	0.512
Age (years)	45.66 ± 10.18	44.07 ± 10.24	45.44 ± 9.91	47.49 ± 10.17	0.008
SBP (mmHg)	123.50 ± 10.89	123.28 ± 10.36	123.36 ± 9.53	123.87 ± 12.60	0.865
DBP (mmHg)	79.11 ± 7.48	79.48 ± 7.15	79.21 ± 6.03	78.66 ± 8.98	0.597
BMI (kg/m^2^)	25.82 ± 4.41	26.60 ± 4.84	25.47 ± 4.21	25.38 ± 4.04	0.018
Waist circumference (cm)	91.83 ± 11.07	92.80 ± 11.64	91.25 ± 10.94	91.43 ± 10.59	0.382
Fasting glucose (mmol/L)	10.20 ± 3.93	9.92 ± 3.45	10.12 ± 4.27	10.57 ± 4.04	0.296
HbA_1_c (%)	11.90 ± 2.49	12.09 ± 2.50	11.74 ± 2.37	11.86 ± 2.58	0.434
Fasting C-peptide (ng/ml)	1.68 ± 1.24	1.67 ± 1.07	1.52 ± 0.93	1.84 ± 1.60	0.060
ALT (U/L) (median (IQR))	20.00 (14.00-35.00)	21.00 (14.00-39.00)	21.00 (14.00-38.00)	18.00 (13.00-31.00)	0.080
AST (U/L) (median (IQR))	17.00 (13.00-27.00)	18.00 (14.00-31.00)	18.00 (13.00-28.00)	16.00 (13.00-23.25)	0.089
GGT (U/L) (median (IQR))	30.00 (19.00-54.60)	32.50 (19.00-57.50)	33.00 (19.25-62.00)	29.00 (13.70-49.25)	0.431
ALB (g/L)	39.98 ± 4.47	40.34 ± 5.14	40.51 ± 3.86	39.09 ± 4.20	0.006
Uric acid (μmol/L)	334.62 ± 113.50	337.90 ± 120.08	325.17 ± 100.01	340.88 ± 119.43	0.401
Creatinine (μmol/L) (median (IQR))	64.90 (55.15-73.70)	61.90 (53.55-69.30)	65.35 (54.95-72.93)	70.75 (57.76-81.10)	<0.001
eGFR (ml/min/1.73m^2^) (median (IQR))	118.47 (103.08-137.48)	125.12 (110.96-142.81)	119.43 (103.23-138.72)	109.62 (93.28-130.21)	<0.001
BUN (mmol/L)	4.27 ± 1.85	2.74 ± 0.64	4.07 ± 0.54	5.99 ± 2.08	<0.001
UACR (median (IQR))	4.84 (2.64-10.92)	4.92 (2.48-10.91)	4.94 (2.87-10.25)	4.58 (2.72-12.15)	0.855
TG (mmol/L) (median (IQR))	1.60 (1.08-2.58)	1.66 (1.08-2.99)	1.58 (1.08-2.55)	1.59 (1.06-2.49)	0.694
TC (mmol/L)	4.82 ± 1.62	4.93 ± 1.43	4.64 ± 1.01	4.89 ± 2.20	0.215
LDL-C (mmol/L)	3.13 ± 0.93	3.17 ± 0.93	3.13 ± 0.95	3.07 ± 0.93	0.629
HDL-C (mmol/L)	1.00 ± 0.28	0.99 ± 0.29	0.98 ± 0.27	1.02 ± 0.28	0.477
BAR	0.11 ± 0.05	0.07 ± 0.01	0.10 ± 0.01	0.16 ± 0.07	<0.001
History of drinking					0.447
Never (case, %)	341, 66.9%	108, 63.5%	114, 67.1%	119, 70.0%	
Current or ever (case, %)	169, 33.1%	62, 36.5%	56, 32.9%	51, 30.0%	
History of smoking					0.662
Never (case, %)	322, 63.1%	108, 63.5%	103, 60.6%	111, 65.3%	
Current or ever (case, %)	188, 36.9%	62, 33.0%	67, 35.6%	59, 31.4%	
baPWV (cm/s)	1469.15 ± 295.82	1406.42 ± 255.04	1462.12 ± 285.45	1538.92 ± 328.85	<0.001
Arterial stiffness, (case, %)	259, 50.8%	69, 40.6%	89, 52.4%	101, 59.4%	0.002

SBP, systolic blood pressure; DBP, diastolic blood pressure; BMI, body mass index; HbA1c, hemoglobin A1c; ALT, alanine aminotransferase; AST, aspartate aminotransferase; GGT, γ-glutamyl transferase; ALB, albumin; BUN, blood urea nitrogen; UACR, urine albumin-to-creatinine ratio; TG, triglyceride; TC, total cholesterol; HDL-C, high-density lipoprotein cholesterol; LDL-C, low-density lipoprotein cholesterol; BAR, blood urea nitrogen to serum albumin ratio; baPWV, brachial-ankle pulse wave velocity

### 3.2 Relationships between BAR and clinical indicators in patients with newly diagnosed with T2DM

As shown in **Table 2**, linear correlation analysis revealed that BAR was positively correlated with age (*r* = 0.222, *p* = 0.001), fasting C-peptide (*r* = 0.206, *p* < 0.001), creatinine (*r* = 0.615, *p* < 0.001), BUN (*r* = 0.950, *p* < 0.001), UACR (*r* = 0.362, *p* < 0.001 ) and baPWV (*r* = 0.226, *p* < 0.001) and negatively correlated with BMI (*r* = -0.089, *p* = 0.045), ALT (*r* = -0.091, *p* = 0.039), AST (*r* = -0.090, *p* = 0.042), ALB (*r* = -0.253, *p* < 0.001) and eGFR(*r* = -0.305, *p* < 0.001) in the total population. Among male patients, BAR was positively associated with age (*r* = 0.199, *p* < 0.001), fasting C-peptide (*r* = 0.189, *p* < 0.001), creatinine (*r* = 0.701, *p* = 0.006), BUN (*r* = 0.947, *p* < 0.001), UACR (*r* = 0.376, *p* < 0.001), HDL-C (*r* = 0.122, *p* = 0.016) and baPWV (*r* = 0.210, *p* < 0.001). However, it was negatively correlated with ALT (*r* = -0.110, *p* = 0.027), AST (*r* = -0.113, *p* = 0.023) and eGFR(*r* = -0.319, *p* < 0.001). In female patients, BAR was positively correlated with age (*r* = 0.371, *p* < 0.001), fasting C-peptide (*r* = 0.286, *p* = 0.003), creatinine (*r* = 0.280, *p* = 0.004), BUN (*r* = 0.937, *p* < 0.001) and baPWV (*r* = 0.334, *p* < 0.001), and negatively correlated with uric acid (*r* = 0.190, *p* = -0.049) and eGFR(*r* = -0.230, *p =* 0.017). Multifactorial stepwise linear regression analysis revealed a significant positive correlation between BAR and baPWV(*β* = 0.187, *p* < 0.001) after adjusting for relevant confounders (**Table 3**).

**Table 2:** Correlation analysis of relationship between lnBAR and characteristics in patients with type 2 diabetes.

Variables	*Whole*		*Male*		*Female*	
	*r*	*p*-value	*r*	*p*-value	*r*	*p*-value
Age	0.222	0.001	0.199	<0.001	0.371	<0.001
SBP	-0.009	0.884	-0.019	0.712	0.018	0.857
DBP	-0.049	0.275	-0.010	0.849	-0.180	0.062
BMI	-0.089	0.045	-0.083	0.097	-0.122	0.211
Waist circumference	-0.029	0.524	-0.088	0.086	0.047	0.633
Fasting glucose	0.072	0.108	0.076	0.130	0.023	0.812
HbA_1_c	-0.046	0.316	-0.063	0.224	0.018	0.854
Fasting C-peptide	0.206	<0.001	0.189	<0.001	0.286	0.003
ALT	-0.091	0.039	-0.110	0.027	-0.072	0.459
AST	-0.090	0.042	-0.113	0.023	-0.048	0.619
GGT	-0.041	0.361	-0.093	0.065	0.052	0.597
ALB	-0.253	<0.001	-0.283	<0.001	-0.165	0.088
Uric acid	0.036	0.423	0.083	0.096	-0.190	0.049
Creatinine	0.615	<0.001	0.701	<0.001	0.280	0.004
eGFR	-0.305	<0.001	-0.319	<0.001	-0.230	0.017
BUN	0.950	<0.001	0.947	<0.001	0.973	<0.001
UACR	0.362	<0.001	0.376	<0.001	0.153	0.199
TG	-0.031	0.489	-0.080	0.112	0.093	0.342
TC	-0.073	0.103	-0.075	0.136	-0.047	0.632
LDL-C	-0.059	0.189	-0.037	0.462	-0.136	0.166
HDL-C	0.085	0.059	0.122	0.016	0.071	0.469
baPWV	0.226	<0.001	0.210	<0.001	0.334	<0.001

SBP, systolic blood pressure; DBP, diastolic blood pressure; BMI, body mass index; HbA1c, hemoglobin A1c; ALT, alanine aminotransferase; AST, aspartate aminotransferase; GGT, γ-glutamyl transferase; ALB, albumin; BUN, blood urea nitrogen; UACR, urine albumin-to-creatinine ratio; TG, triglyceride; TC, total cholesterol; HDL-C, high-density lipoprotein cholesterol; LDL-C, low-density lipoprotein cholesterol; BAR, blood urea nitrogen to serum albumin ratio; baPWV, brachial-ankle pulse wave velocity.

**Table 3:** Relationships between baPWV and various covariates including BAR, according to multiple stepwise linear regression analyses.

Variables	Unstandardized coefficients (95% CI)	Standardized coefficients	*p*-value
Age	10.525 (8.109, 12.941)	0.374	<0.001
SBP	8.687 (6.498, 10.875)	0.337	<0.001
HbA1c	-18.788 (-28.691, -8.884)	-0.163	<0.001
ALT	-0.835 (-1.552,-0.119)	-0.101	0.022
BAR	958.651 (518.429, 1398.874)	0.187	<0.001

SBP, systolic blood pressure; HbA1c, hemoglobin A1c; UACR, urine albumin-to-creatinine ratio; BAR, blood urea nitrogen to serum albumin ratio.

### 3.3 Association of BAR with arterial stiffness in patients with newly diagnosed with T2DM

Arterial stiffness (baPWV ≥1400 cm/s) was assessed in relation to BAR using multivariable binary logistic regression analysis (**Table 4**). Three models were constructed to evaluate this association. As a continuous variable, each unit increase in the lnBAR was significantly associated with a greater risk of arterial stiffness (OR = 2.845, 95% CI: 1.745- 4.639, *p* < 0.001) in Model 1, with no adjustment for covariates. After adjusting for age and sex in Model 2, the lnBAR remained significantly associated with arterial stiffness (OR = 2.483, 95% CI: 1.484- 4.153, *p* = 0.001). After adjusting for age, sex, systolic blood pressure, diastolic blood pressure, BMI, WC, fasting glucose, fasting C-peptide, HbA1c, ALT, AST, GGT, creatinine, uric acid, TG, TC, HDL-C, and LDL-C, the associations between lnBAR and arterial stiffness remained significant (OR = 3.452, 95% CI: 1.586- 7.513, *p* = 0.002). When analyzed categorically, the T2 and T3 BAR groups presented a significantly greater risk of arterial stiffness than the T1 subgroup (T2: OR = 1.608, 95% CI: 1.047- 2.471, *p* = 0.030, T3: OR = 2.143, 95% CI: 1.390- 3.303, *p* = 0.001). In Model 2, the T3 subgroup had a greater risk than the T1 subgroup (OR = 1.914, 95% CI: 1.209- 3.029, *p* = 0.006). Following full adjustment, both the T2 (OR = 1.915, 95% CI: 1.106- 3.609, *p* = 0.044)) and T3 (OR = 2.064, 95% CI: 1.051- 4.054, *p* = 0.035) groups presented persistently elevated risks compared with the T1 group.

**Table 4:** Multivariable binary logistic regression for the association between BAR with arterial stiffness.

Variables	Model 1 OR (95%CI), *p*-value	Model 2 OR (95%CI), *p*-value	Model 3 OR (95%CI), *p*-value
baPWV (cm/s)			
lnBAR	2.845 (1.745, 4.639), <0.001	2.483 (1.484, 4.153), 0.001	3.452 (1.586, 7.513), 0.002
BAR			
T1	Ref	Ref	Ref
T2	1.608 (1.047, 2.471), 0.030	1.553 (0.987, 2.443), 0.057	1.915 (1.016, 3.609), 0.044
T3	2.143 (1.390, 3.303), 0.001	1.914 (1.209, 3.029), 0.006	2.064 (1.051, 4.054), 0.035
*p* for trend	0.015	<0.001	<0.001

lnBAR, BAR levels were analyzed as naturally logarithmically transformed values.

BAR, blood urea nitrogen to serum albumin ratio; baPWV, brachial-ankle pulse wave velocity.

Model 1: unadjusted model.

Model 2: adjusted for age, sex.

Model 3: additionally adjusted for SBP, DBP, BMI, waist circumference, history of drinking, history of smoking, fasting glucose, fasting C-peptide, HbA1c, ALT, AST, GGT, Uric acid, UACR, eGFR, TG, TC, HDL-C, LDL-C.

### 3.4 Stratified analysis of BAR and arterial stiffness

The association between BAR and arterial stiffness was assessed by stratifying the covariates including age, sex, and BMI. The positive association between BAR and baPWV remained consistent when the data were divided into subgroups according to sex (male and female) and BMI (<25 kg/m² and ≥25 kg/m²)(*p* for interaction >0.05). An interaction effect for the association between BAR and baPWV was present between age groups, and the positive association between BAR and baPWV was found to be more significant in the <50 years subgroup than in the ≥50 years subgroup (**Table 5**).

**Table 5:** Stratified analysis of the association between BAR and arterial stiffness.

Variables	Adjusted II OR(95%CI)	*p*-value	*p* for interaction
Stratified by Gender			0.362
Female	20.007(1.421, 281.780)	0.026	
Male	3.002(1.386, 6.503)	0.005	
Stratified by Age			0.011
<50 years	3.402(1.378, 8.397)	0.008	
≥50 years	2.532(0.746, 8.598)	0.136	
Stratified by BMI			0.495
<25kg/m^2^	3.729(1.177, 11.810)	0.025	
≥25kg/m^2^	3.212(1.218, 8.471)	0.018	

Adjusted for age, sex, SBP, DBP, BMI, waist circumference, history of drinking, history of smoking, fasting glucose, fasting C-peptide, HbA1c, ALT, AST, GGT, Uric acid, UACR, eGFR, TG, TC, HDL-C, LDL-C.

### 3.5 The cut-off BAR value to indicate arterial stiffness analyzed by ROC

**Figure 1:**
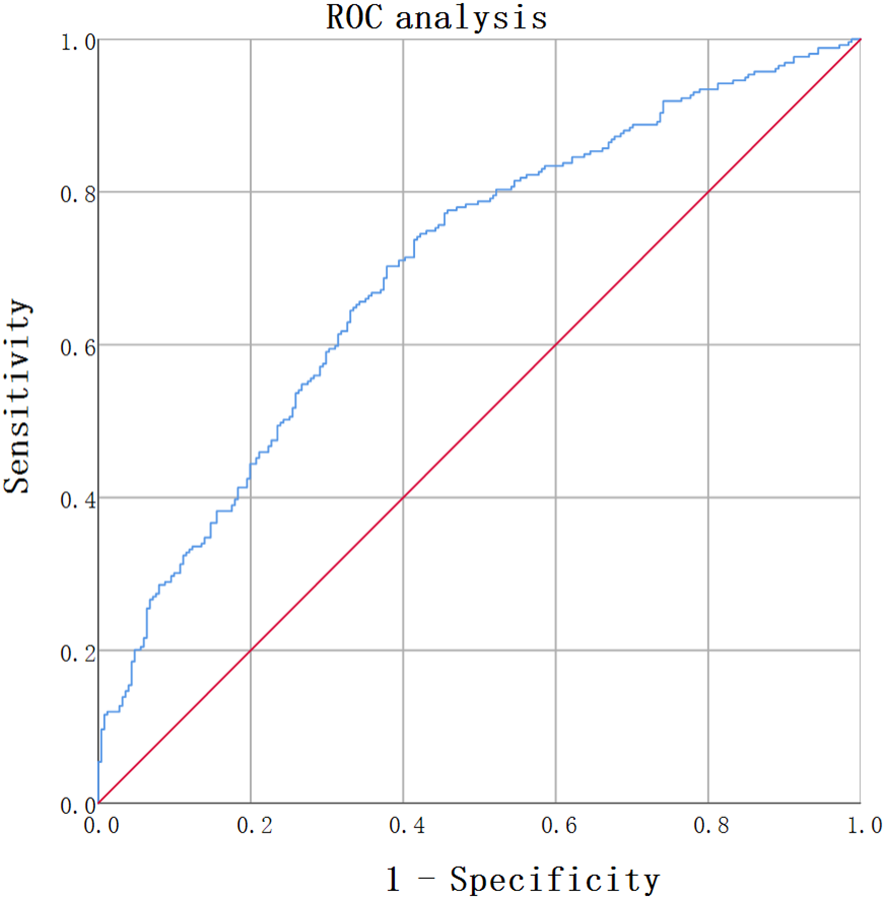
ROC analysis of BAR for predicting arterial atiffness in newly diagnosed T2DM. ROC analysis was used to identify the optimal cut-off value of BAR to predict increased arterial stiffness in T2DM. The AUC was 0.699(95% CI: 0.654-0.744). The optimal BAR cut-off was 0.1089. Youden index was 0.325, with a sensitivity of 70.3%, and a specificity of 62.2%.

The optimal cut-off value of BAR to predict increased arterial stiffness (defined as baPWV≥1400cm m/s) in newly diagnosed with T2DM was determined through ROC curve analysis. The value that maximized the Youden Index was selected as the optimal threshold. From our analysis of 510 patients, the optimal BAR cut-off was 0.1089, with an area under the curve (AUC) of 0.699(95% CI:0.654- 0.744), with a sensitivity of 70.3% and a specificity of 62.2% (**Figure 1**).

## 4 Discussion

In this study, we investigated the relationship between arterial stiffness, as quantified by baPWV and BAR, in patients newly diagnosed with T2DM. To our knowledge, this study is the first to identify a positive association between BAR and arterial stiffness in T2DM patients, even after adjusting for relevant potential confounders. In each subgroup, the positive association between BAR and baPWV was consistently maintained, as shown by further stratified analyses. Thus, these findings suggest that BAR may be useful in assessing early arterial stiffness in people with T2DM.

Research has consistently shown that there is a significant connection between arterial stiffness and atherosclerotic diseases, including myocardial infarction, heart failure, peripheral vascular disease, and stroke (20–22). Additionally, widespread cardiovascular risk factors such as diabetes, aging, hypertension, obesity, and chronic kidney disease can increase arterial stiffness and decrease elasticity (23). Pulse wave velocity (PWV) is an important index for assessing arterial stiffness and can be characterized by carotid-femoral PWV (cfPWV), brachial-ankle PWV (baPWV), aortic PWV (aPWV), femoral-ankle PWV (faPWV), and carotid-radial PWV (crPWV) according to the pulse wave recording area. Compared with other parameters of arterial stiffness, such as the CAI, CAVI, and CAP, PWV is the most reliable indicator of CVD and is strongly correlated with CVD events and their risk factors, such as hypertension, obesity, and diabetes (9, 24). An increase in arterial stiffness is positively correlated with an increased risk of cardiovascular events and all-cause mortality among T2DM patients (25). The baPWV is a clinically validated, simple, and cost-effective measure for evaluating arterial stiffness. Prospective clinical studies have demonstrated that arterial stiffness, quantified by baPWV, is a predictor of all-cause and cause-specific mortality in individuals with T2DM (8, 26). Research has indicated that an elevated baPWV acts as an independent predictor of adverse prognosis in individuals with ASCVD (27). A 1-SD increase in baPWV raises the likelihood of cardiovascular events by 1.41 times (27). An improvement in arterial stiffness was related to a 43% decrease in the risk of primary composite events, which included stroke, myocardial infarction, and all-cause mortality (28). Research has further indicated that arterial stiffness is an independent risk factor for heart failure. Notably, for every 359 cm/s increase in baPWV, the risk of heart failure increases by 10% over an average period of 5.53 years (20). Recent research has indicated a potential bidirectional connection between the onset of diabetes and arterial stiffness. The Chinese Kailuan Study cohort demonstrated that an increased baPWV significantly elevates the risk of developing diabetes, suggesting that arterial stiffness may act as an early pathological contributor to the development of the disease (29). Further study suggested that aortic stiffness may impair glucose metabolic homeostasis through microvascular damage to the pancreas, liver, and skeletal muscle, potentially serving as an early predictive marker of diabetes (30). Therefore, early assessment of arterial stiffness could be beneficial for predicting T2DM and diabetes-related macrovascular diseases. The identification of simple biomarkers to predict arterial stiffness in clinical settings remains a focal point of research in the field of metabolic diseases.

BUN and ALB are widely used biochemical markers that are easily accessible in routine clinical practice and offer a cost-effective means of assessment. Elevated BUN levels have been shown to be associated with a heightened risk of developing diabetes and its microvascular complications (31, 32), along with a positive, non-linear relationship with the risk of all-cause and cardiovascular mortality in individuals with T2DM (11). Elderly patients with low ALB levels are more likely to have cardiovascular disease, and even those within low-normal ALB level may also be at higher risk of CVD and mortality (33). BAR acts as an indicator of variations in BUN and ALB levels, offering insights into nutritional health, protein metabolism, and the function of the liver and kidney. It serves as an independent predictor of adverse prognosis and short-term mortality in various critical illnesses including pneumonia, sepsis, renal insufficiency, and CVD (14–17, 34–36). Recent studies have identified BAR as a significant prognostic marker of chronic diseases. In a cohort of healthy individuals undergoing physical examinations, a significant positive correlation was identified between BAR and cerebral small vessel disease, including all its subtypes, in a dose-response relationship (37). Moreover, a recent study has identified a significant positive correlation between elevated BAR and increased risks of CVD (OR 1.09), cardiovascular mortality (OR 1.13), and all-cause mortality (OR 1.12) in patients with diabetes (38). In our study, 510 patients with newly diagnosed T2DM were stratified according to BAR tertiles. The group in the highest BAR group presented increased age, blood creatinine and BUN levels. Additionally, the highest BAR group had a greater proportion of patients with arterial stiffness than the other groups. Regardless of whether it was treated as a continuous or categorical variable, BAR was positively correlated with elevated baPWV after adjusting for relevant factors. These findings are consistent with the hypothesis that BAR may interact with various metabolic factors influencing cardiovascular disease and could be a useful marker for the early differentiation of patients with newly diagnosed T2DM with or without arterial stiffness. The stratified analyses indicated that the independent association between the BAR and elevated baPWV in different groups divided by sex and BMI remained consistent. Nevertheless, the limited sample size of the female group may result in imprecise estimated values. These results should be validated in larger, adequately powered studies. Stratified analysis also revealed that the association between BAR and baPWV was more significant in younger patients newly diagnosed with T2DM who were <50 years old. This finding suggests that BAR may be a more prominent correlation factor of arterial stiffness in younger patients with T2DM. Previous studies has been demonstrated that arterial stiffness is more readily improved in young individuals when a healthy lifestyle is adopted (28). In contrast, arterial stiffness in older patients may be influenced by aging, multiple concurrent diseases, and other factors beyond liver and kidney function, thereby potentially explaining the observed attenuation of the association between BAR and baPWV in this subgroup.

The pathophysiological mechanisms connecting BAR and arterial stiffness in individuals with T2DM remain incompletely understood. Previous research has demonstrated a significant association between BAR and type 2 diabetic retinopathy, nephropathy, and macrovascular diseases (17, 38, 39). It was hypothesized that this association was attributable to the effects of the two principal components that constitute BAR. In the hyperglycemic state of T2DM, the body experiences hemodynamic abnormalities, renal function impairment, inflammation, oxidative stress, and endothelial dysfunction. These conditions lead to changes in BUN and ALB levels, promote arterial stiffness, and increase cardiovascular risk and adverse prognosis in patients with diabetes. First, BUN is a critical parameter for evaluating renal function. Elevated BUN levels signify increased protein catabolism and impaired renal function (40). Arterial stiffness and chronic kidney disease may share common pathological pathways that facilitate their co-occurrence and progression (23). The Framingham study established a significant correlation between PWV and the onset of proteinuria (41). Early vascular dysfunction, indicated by a baPWV exceeding the 90th percentile, was significantly associated with proteinuria alone, impaired renal function alone, and the combined risk of proteinuria and renal dysfunction (42). Second, existing research has demonstrated that inflammation is an important factor in the development of diabetes and its related complications (39). Elevated BUN levels have been linked to inflammation, oxidative stress, and compromised endothelial function (43, 44). Urea can induce endoplasmic reticulum stress by inactivating the anti-atherogenic enzyme PGI2 synthase, thereby impairing endothelial function by inhibiting of cell proliferation and inducing endothelial-mesenchymal transition (45, 46). Additionally, urea induces the production of oxygen free radicals from endothelial progenitor cells (EPCs), leading to impaired EPCs function and the development of cardiovascular disease (47). Plasma ALB fulfills several physiological functions, including reducing inflammation, preventing oxidative stress, preservation of vascular endothelial integrity. Research indicates that ALB can attenuate the expression of VCAM-1 induced by TNF-α, thereby inhibiting the onset of vascular inflammation (48). Furthermore, ALB acts as a scavenger of oxygen-free radicals, inhibits the activity of angiotensin-converting enzymes, reduces the sensitivity of blood vessels to nitric oxide, and reduces apoptosis in endothelial cells (49, 50). Finally, elevated BUN levels may serve as indirect markers of a hypercoagulable state, thereby facilitating the onset of diabetic complications. Serum albumin also contributes to the stability of the circulatory system through its anticoagulant, antithrombotic, and antiplatelet aggregation properties (51).

This study encountered various limitations. Firstly, the cross-sectional design of the study precludes the establishment of causal relationships between BAR and baPWV, and the possibility of reverse causality cannot be dismissed. Secondly, despite adjustments for multiple confounding variables, residual confounding, such as that related to lifestyle factors or unmeasured inflammatory markers, may persist. Thirdly, the single-center design and the homogeneous Chinese cohort limit the generalizability of the findings to other populations. Fourthly, the inclusion of only newly diagnosed T2DM patients may restrict the applicability of the results to individuals with a longer disease duration or those undergoing treatment. Finally, the absence of longitudinal data precludes the assessment of BAR’s predictive value for the progression of arterial stiffness. Consequently, it is essential to conduct more prospective randomized controlled trials to substantiate the conclusions of this study.

## 5 Conclusions

In conclusion, our findings indicate that BAR is positively and independently associated with arterial stiffness, as determined by baPWV, in Chinese patients with newly diagnosed T2DM. These results imply that BAR levels could play a role in improving the management of cardiovascular complications in patients with newly diagnosed T2DM, particularly those younger than 50 years.

## Data Availability

The raw data supporting the conclusions of this article will be made available by the authors, without undue reservation.
