# Supplementary material for: Association of blood urea nitrogen to serum albumin ratio with arterial stiffness in type 2 diabetes patients: a Chinese cross-sectional study
Source: Front Endocrinol (Lausanne). 2025 Oct 8;16:1663831. doi: 10.3389/fendo.2025.1663831 (PMC12540143; doi:10.3389/fendo.2025.1663831)
Supplement: Supplementary file 1 [file Table1.docx]

**Supplement Table 1: Multivariable binary logistic regression for the association between relative markers with arterial stiffness**

|  | Model 3 OR(95%CI), *p*-value |
| --- | --- |
| Age | 1.083(1.048, 1.120), < 0.001 |
| Sex(Female) | 1.088(0.494, 2.396), 0.834 |
| SBP | 1.075(1.040, 1.111), < 0.001 |
| DBP | 1.016(0.974, 1.060), 0.459 |
| BMI | 1.141(1.006, 1.295), 0.040 |
| waist circumference | 0.957(0.910, 1.006), 0.083 |
| history of drinking | 0.893(0.495, 1.612), 0.707 |
| history of smoking | 0.832(0.456, 1.516), 0.547 |
| fasting glucose | 1.077(1.002, 1.158), 0.043 |
| fasting C-peptide | 1.363(0.992, 1.873), 0.056 |
| HbA1c | 0.826(0.731, 0.933), 0.002 |
| ALT | 0.984(0.969, 1.000), 0.046 |
| AST | 1.010(0.986, 1.035), 0.402 |
| GGT | 1.002(0.998, 1.006), 0.337 |
| Uric acid | 1.000(0.997, 1.002), 0.819 |
| UACR | 1.003(0.998, 1.009), 0.248 |
| eGFR | 1.010(1.000, 1.020), 0.051 |
| TG | 0.884(0.714, 1.094), 0.256 |
| TC | 1.317(0.642, 2.701), 0.452 |
| HDLC | 0.796(0.268, 2.363), 0.681 |
| LDLC | 0.800(0.397, 1.612), 0.533 |
| lnBAR | 3.452(1.586, 7.513), 0.002 |

lnBAR, BAR levels were analyzed as naturally logarithmically transformed values.

SBP, systolic blood pressure; DBP, diastolic blood pressure; BMI, body mass index; HbA1c, hemoglobin A1c; ALT, alanine aminotransferase; AST, aspartate aminotransferase; GGT, γ-glutamyl transferase; UACR, urine albumin-to-creatinine ratio; TG, triglyceride; TC, total cholesterol; HDL-C, high-density lipoprotein cholesterol; LDL-C, low-density lipoprotein cholesterol; lnBAR, naturally logarithmically transformed blood urea nitrogen to serum albumin ratio

**Supplement Table 2: Multicollinearity was assessed for variables included in the multivariable linear regression model.**

| Variables | Standardized  coefficients | *p*-value | *VIF* |
| --- | --- | --- | --- |
| Age | 0.374 | <0.001 | 1.026 |
| SBP | 0.337 | <0.001 | 1.004 |
| HbA1c | -0.163 | <0.001 | 1.026 |
| ALT | -0.101 | 0.022 | 1.037 |
| BAR | 0.187 | <0.001 | 1.020 |
| DBP | 0.027 | 0.606 | 1.453 |
| BMI | 0.039 | 0.407 | 1.202 |
| Waist circumference | 0.031 | 0.495 | 1.127 |
| Fasting glucose | 0.088 | 0.053 | 1.119 |
| Fasting C-peptide | 0.025 | 0.594 | 1.214 |
| AST | 0.069 | 0.444 | 4.387 |
| GGT | 0.059 | 0.210 | 1.197 |
| eGFR | 0.031 | 0.533 | 1.365 |
| Uric acid | -0.013 | 0.779 | 1.175 |
| UACR | 0.028 | 0.533 | 1.365 |
| TG | -0.040 | 0.369 | 1.047 |
| TC | -0.050 | 0.250 | 1.029 |
| LDL-C | -0.010 | 0.817 | 1.051 |
| HDL-C | -0.026 | 0.545 | 1.019 |

SBP, systolic blood pressure; HbA1c, hemoglobin A1c; ALT, alanine aminotransferase; BAR, blood urea nitrogen to serum albumin ratio; DBP, diastolic blood pressure; BMI, body mass index; AST, aspartate aminotransferase; GGT, γ-glutamyl transferase; UACR, urine albumin-to-creatinine ratio; TG, triglyceride; TC, total cholesterol; LDL-C, low-density lipoprotein cholesterol; HDL-C, high-density lipoprotein cholesterol

**Supplement Table 3: Multivariable binary logistic regression included different variables**

|  | **models with BAR** | **models with BUN alone** | **models with albumin alone** | **models with BUN and albumin** | |
| --- | --- | --- | --- | --- | --- |
|  | lnBAR | lnBUN | ALB | lnBUN | ALB |
| Model 3 OR(95%CI) | 3.452(1.586, 7.513) | 2.751(1.666, 6.445) | 1.089(1.004, 1.851) | 2.778(1.676, 6.517) | 0.984(0.925, 1.048) |
| *p*-value | 0.002 | 0.001 | 0.034 | 0.001 | 0.120 |

adjusted for age, sex, SBP, DBP, BMI, waist circumference, history of drinking, history of smoking, fasting glucose, fasting C-peptide, HbA1c, ALT, AST, GGT, Uric acid, UACR, eGFR, TG, TC, HDL-C, LDL-C.

**Supplement Table 4: Sensitivity analysis for the association between BAR with baPWV≥1500cm/s**

|  | Model 1 OR(95%CI), *p*-value | Model 2 OR(95%CI), *p*-value | Model 3 OR(95%CI), *p*-value |
| --- | --- | --- | --- |
| baPWV（cm/s） |  |  |  |
| lnBAR | 3.155(1.767, 5.635), <0.001 | 2.497(1.330, 4.685), 0.004 | 4.126(1.585, 10.741)，0.004 |
| BAR |  |  |  |
| T1 | Ref | Ref | Ref |
| T2 | 1.548(0.976 2.452), 0.063 | 1.465(0.896, 2.395), 0.128 | 1.712(0.834, 3.514), 0.143 |
| T3 | 2.340(1.487, 3.682), <0.001 | 2.050(1.260, 3.335), 0.004 | 2.559(1.208, 5.421), 0.014 |
| *P* for trend | <0.001 | <0.001 | <0.001 |

lnBAR, BAR levels were analyzed as naturally logarithmically transformed values.

BAR, blood urea nitrogen to serum albumin ratio; baPWV, brachial-ankle pulse wave velocity.

Model 1: unadjusted model.

Model 2:adjusted for age, sex.

Model 3: additionally adjusted for SBP, DBP, BMI, waist circumference, history of drinking, history of smoking, fasting glucose, fasting C-peptide, HbA1c, ALT, AST, GGT, Uric acid, UACR, eGFR, TG, TC, HDL-C, LDL-C.
